# A novel, simple, and dedicated device for endoscopic mucosal defect closure

**DOI:** 10.1002/deo2.98

**Published:** 2022-03-09

**Authors:** Yohei Nose, Kohei Takizawa, Kazuo Shiotsuki, Tsuyoshi Yamaguchi, Masaomi Agatsuma, Shun Nitta, Kotaro Yamashita, Takuro Saito, Koji Tanaka, Kazuyoshi Yamamoto, Tomoki Makino, Tsuyoshi Takahashi, Yukinori Kurokawa, Hidetoshi Eguchi, Yuichiro Doki, Kiyokazu Nakajima

**Affiliations:** ^1^ Department of Next Generation Endoscopic Intervention (Project ENGINE) Graduate School of Medicine Osaka University Osaka Japan; ^2^ Department of Gastroenterological Surgery Graduate School of Medicine, Osaka University Osaka Japan; ^3^ Division of Endoscopy Shizuoka Cancer Center Shizuoka Japan; ^4^ Department of Gastroenterology and Endoscopy Sapporo Kinentou hospital Hokkaido Japan; ^5^ Hakko Co. Ltd. Nagano Japan

**Keywords:** device development, endoscopic submucosal dissection, mucosal defect closure

## Abstract

**Objectives:**

Endoscopic submucosal dissection (ESD) has become popular, but complications such as postoperative bleeding remain an issue. Although some methods of closing a mucosal defect with a snare and clips have been reported to be effective and safe, the snare is not a dedicated device, and the procedure is difficult and time‐consuming. We aimed to find an alternative method for defect closure after ESD by developing a dedicated device.

**Methods:**

We have improved five prototypes. The load on the stopper when starting to tighten and loosen a loop and the maximum load on the stopper and the movement distance of the thread when sliding the stopper were measured five times for each prototype. With the 5th prototype, we finalized the design and named it FLEXLOOP. Additionally, the material and shape of the outer tube were improved. Then, the usability of FLEXLOOP was evaluated in pigs. The operation time for closing mucosal defects with the snare or FLEXLOOP was measured five times.

**Results:**

We made FLEXLOOP, which had a lower load when sliding and a higher load when loosening than the snare. The improvement of the outer tube significantly reduced the load on the sheath when sliding it. We confirmed the feasibility of mucosal defect closure with FLEXLOOP in pigs. The median operation time was 563 s (range 340–679 s) with the snare and 355 s (range 303–455 s) with FLEXLOOP (*p *= 0.047).

**Conclusions:**

FLEXLOOP can be a promising option for defect closure after ESD.

## INTRODUCTION

With the recent development of gastrointestinal endoscopy, endoscopic submucosal dissection (ESD) has been increasingly performed for the treatment of early gastric cancer (EGC).^1^ Compared to surgery, endoscopic resection is less invasive and allows patients to return to society earlier, which also has great benefits for reducing medical expenses. For these reasons, ESD has rapidly become popular in recent years, but the prevention and reduction of complications due to ESD have become other issues. One of the major complications in gastric ESD is postoperative bleeding, which was reported to be observed in 4.4% of patients.^2^ In addition, with the increase in the aging society, ESD is increasingly likely to be performed in elderly patients with concomitant medical conditions, particularly heart conditions and cerebrovascular disease. Many of these patients are on long‐term antithrombotic therapy (antiplatelet agents or anticoagulants). Antithrombotic therapy is a well‐known independent risk factor for bleeding after ESD for EGC,^3^, ^4^, ^5^, ^6^ and the rate of postoperative bleeding increases to 23.3% in patients treated with antithrombotic therapy.^7^

To prevent post‐ESD bleeding, various technical methods have been described.^8^, ^9^, ^10^, ^11^ Most importantly, a novel method of closing mucosal defects using a ligating device (HX‐20Q‐1; Olympus Medical, Tokyo, Japan) with a detachable snare (MAJ‐254, MAJ‐340; Olympus Medical), which is usually used to ligate the stalk of the polyp, and endoclips (HX‐610; Olympus Medical) has been reported to be effective and safe.^12^, ^13^, ^14^, ^15^, ^16^, ^17^, ^18^, ^19^ However, the conventional snare is not a dedicated device for mucosal defect closure after ESD, and the problem is that the procedure is technically complicated, difficult, and time‐consuming. Therefore, we sought to determine whether the structure of the ENDOLOOP Ligature (Ethicon Inc, New Brunswick, NJ, USA) used in laparoscopic surgery^20^ could be applied to the closure device for flexible endoscopy. The purpose of this study was to find an alternative method of mucosal defect closure after ESD by developing a new dedicated closure device called “FLEXLOOP” in collaboration with a company.

## METHODS

### Prototypes

**FIGURE 1:**
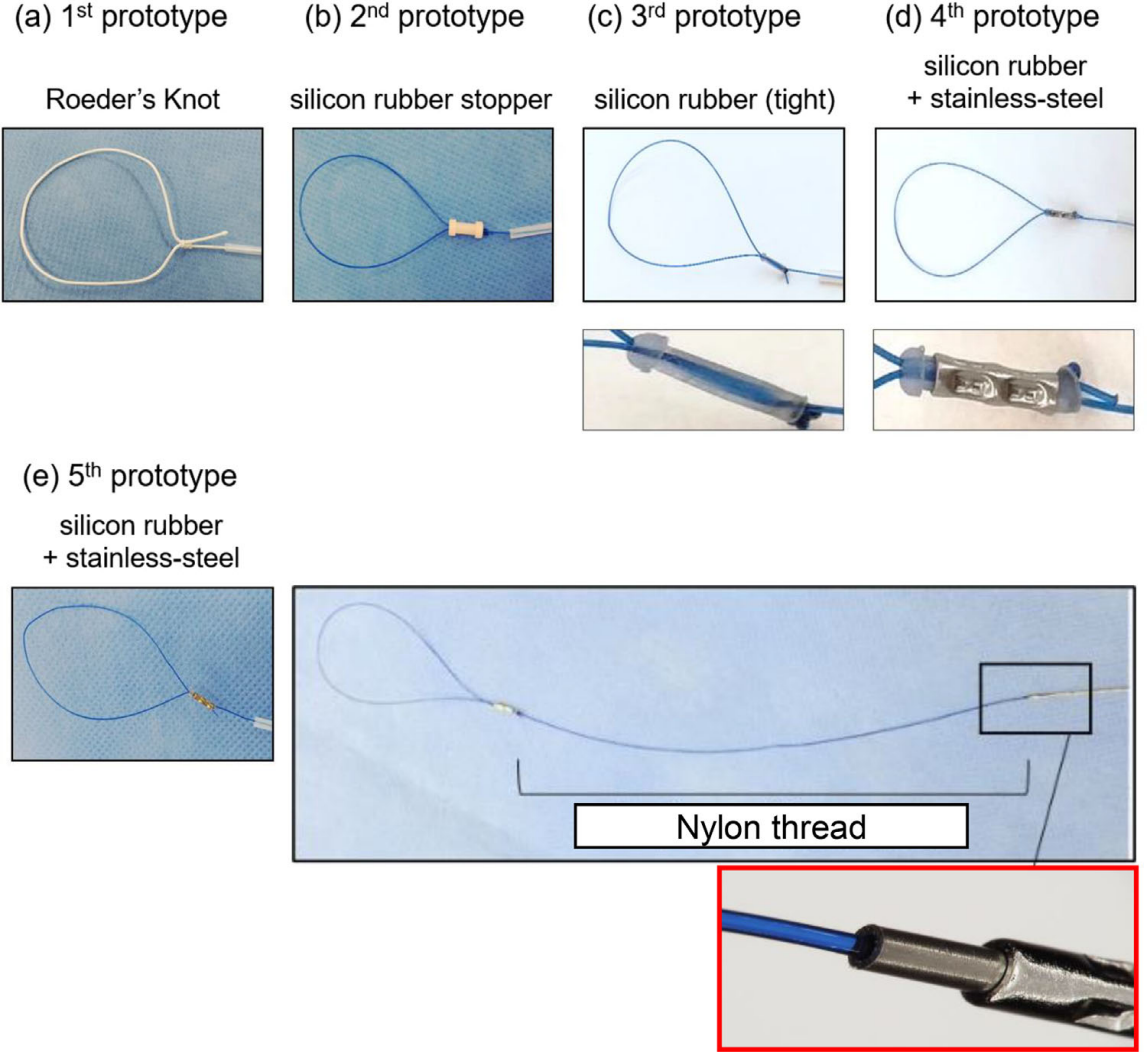
(a) The 1st prototype: The silk thread, which has a loop with a Roeder's knot, was inserted into the outer sheath. (b) The 2nd prototype: The nylon thread, which has a loop‐shaped tip and a silicon rubber stopper attached to the knot, was inserted into the outer sheath. (c) The 3rd prototype: The shape of the stopper was changed to a tighter conformation. (d) The 4th prototype: The stopper was made of silicon rubber with a stainless steel pipe for tight adherence. (e) The 5th prototype: The nylon thread and a stainless steel wire were joined at their midpoints

We have improved five prototypes over the generations, as shown below. The 1st prototype was made of silk thread with a Roeder knot at the distal end of the suture, with reference to the ENDOLOOP Ligature used in laparoscopic surgery (Figure 1a). The 2nd prototype was made of nylon thread (monofilament; 2‐0) with a loop‐shaped tip (loop diameter: 35 mm) and a silicon rubber stopper attached to the knot, which was inserted into the unique outer sheath (inner diameter: 1.3 mm, outer diameter: 2.5 mm, length: 1800 mm) (Figure 1b). In addition, the shape of the stopper was changed to a tighter conformation in the 3^rd^ prototype (Figure 1c). Then, in the 4th prototype, the stopper was made of silicon rubber with a stainless steel pipe for tight adherence (Figure 1d). In the 5th prototype, the nylon thread and a stainless steel wire were joined at their midpoints (Figure 1e). With this last prototype, we finalized the design and named it FLEXLOOP.

**FIGURE 2:**
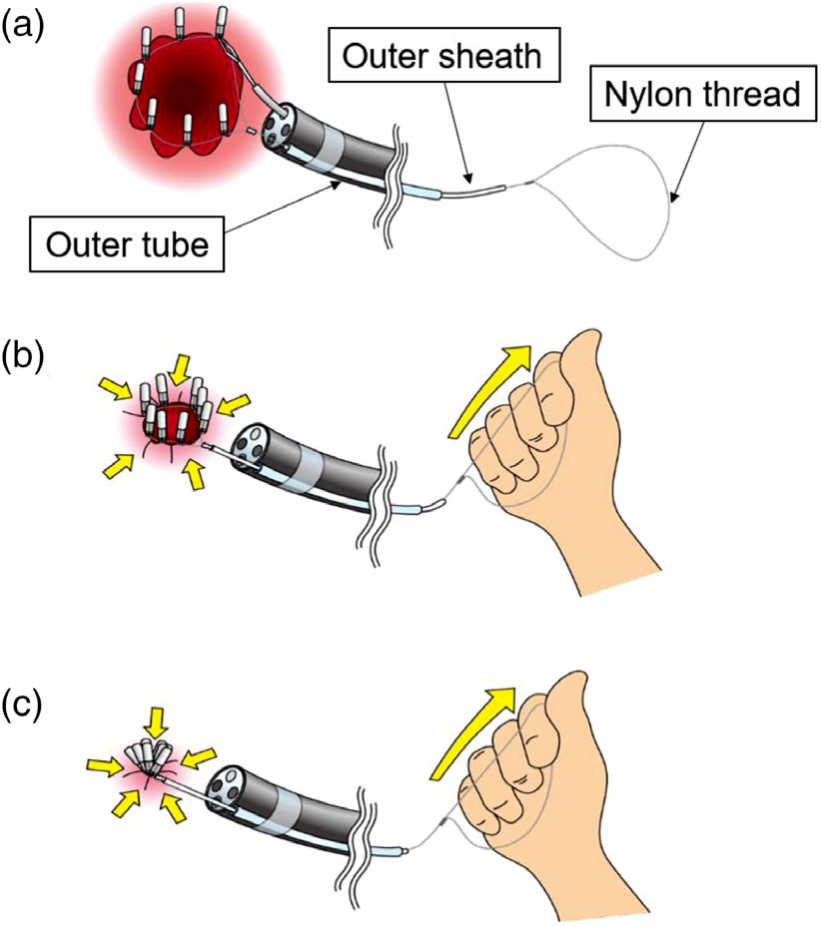
The actual usage: (a) The loop was placed around the defect and fixed with endoclips. (b, c) The defect was then closed by tightening the loop

These prototypes were designed so that the loop at the tip would be tightened when the thread protruding from the back of the outer sheath was pulled and the stopper was pushed with the sheath. Also, during operation, the stopper could only move in the direction of tightening the loop. The actual usage procedure was as follows: Prior to endoscopy, the outer tube was externally attached to a flexible single‐channel gastroscope (GIF‐Q260J; Olympus Medical) with scotch tapes in four or five places. First, the endoscope with the outer tube was advanced into the stomach, the loop with the outer sheath was inserted through the outer tube, and endoscopic clips were inserted through the endoscope channel. Next, the loop was placed around the mucosal defect and fixed with hooked clips (Figure 2a); the mucosal defect was then closed by tightening the fixed loop (Figure 2b,c). Finally, the thread was cut with endoscopic scissors (FS‐3L‐1; Olympus Medical) to complete the procedure.

### Bench testing

First of all, we performed the following bench testings to obtain the benchmark date of the currently available ligating device with the snare (HX‐400U‐30; Olympus Medical). Then, we evaluated and improved prototypes by referring to the benchmark measurements obtained above. The bench testings we conducted were as follows: (a) the load on the stopper when starting to tighten the loop, (b) the load on the stopper when starting to loosen the loop, and (c) the maximum load on the stopper and the movement distance of the thread when sliding the stopper. Lower load on the stopper when starting meant the device was easy to squeeze, and higher load on the stopper when loosening meant the device was tight and kept closure force.

**FIGURE 3:**
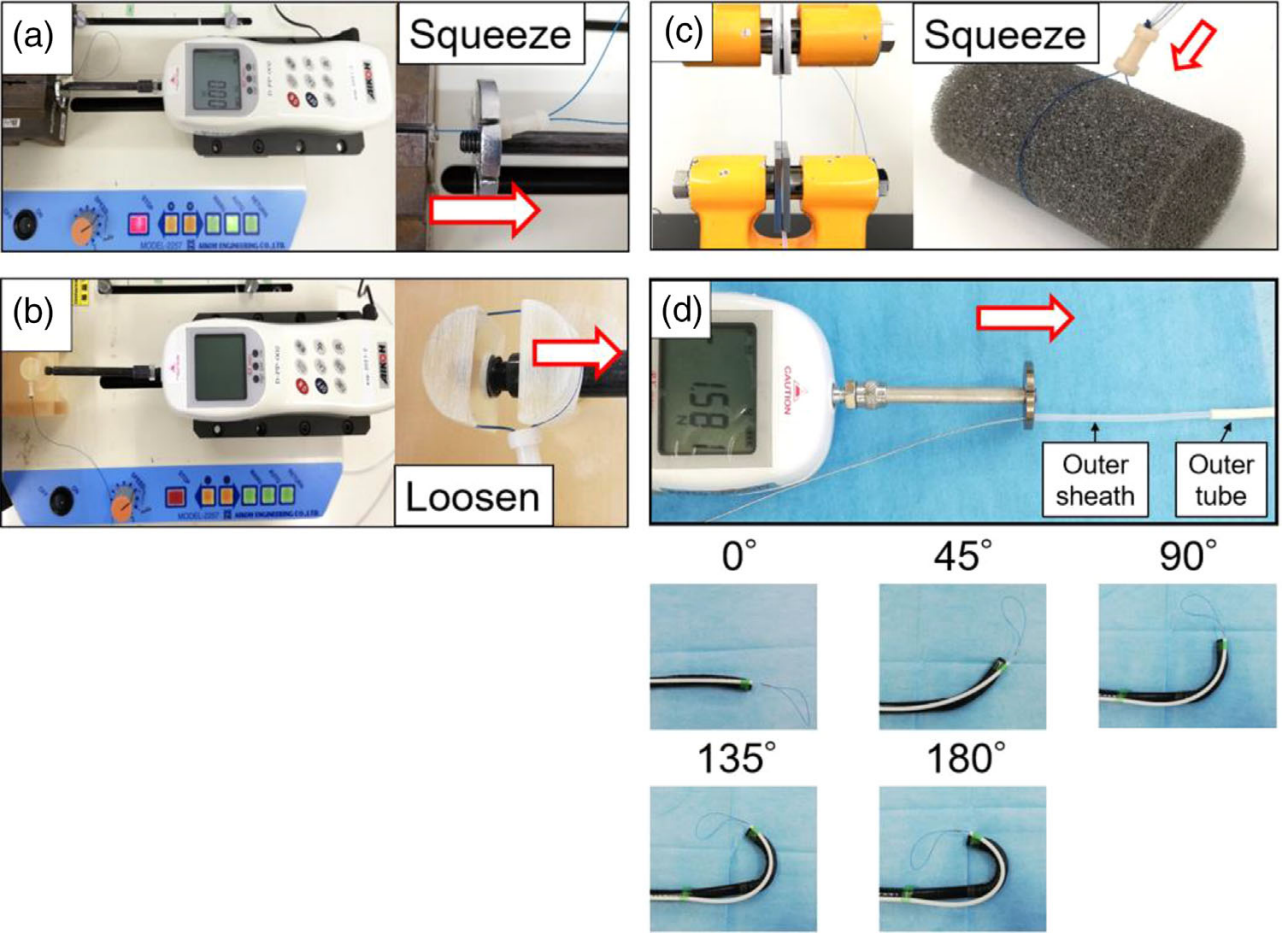
The load on the stopper when starting to slide (a) and loosen the loop (b) was measured. (c) The maximum load on the stopper and the distance of thread movement were measured. (d) The load on the outer sheath when starting to slide it in polyvinyl chloride (PVC) or polyamide/polyurethane tube was measured under the condition of bending the endoscope tip from 0 to 180°

The load on the stopper when starting to tighten the loop was measured by setting each prototype on a test stand (Motorized Stand MODEL‐2257; AIKOH ENGINEERING, Osaka, Japan) and pulling it at 100 mm/min with a measuring instrument (Digital Force Gauge RX‐10; AIKOH ENGINEERING) (Figure 3a). In addition, the loop was applied to a 15 mm cylinder that was set on a test stand without loosening, and the half face of the cylinder was slid at 100 mm/min to measure the load on the stopper when starting to loosen the loop (Figure 3b). Next, we attached the loop to a urethane sponge (F2; INOAC, Aichi, Japan) with a diameter of 30 mm and applied tension to the stopper at 100 mm/min with a measuring instrument (TENSILON RTG‐1310; A&D, Tokyo, Japan) to measure the maximum load on the stopper and the distance of the thread traveled when the diameter of the sponge was reduced from 30 to 20 mm by squeezing (Figure 3c). Finally, the material and shape of the outer tube were changed from polyvinyl chloride (PVC) with an inner diameter of 3.4 mm, an outer diameter of 5.1 mm, and a length of 1600 mm to polyamide/polyurethane with an inner diameter of 2.8 mm, an outer diameter of 3.8 mm, and a length of 1600 mm to improve the operability when inserting and removing the outer sheath. Each outer tube was attached to the endoscope tip, and the load on the sheath when starting to slide the sheath was measured with the measuring instrument under the condition of bending the endoscope tip from 0 to 180° (Figure 3d). All these parameters were measured five times each, and the median values are presented. The unit of measurement for these experiments was the newton (N).

### Animal studies

All procedures were conducted using 3‐month‐old female pigs (average weight of 35 kg) under general anesthesia. Each pig was humanely euthanized upon completion of the experiment. Board‐certified surgeons conducted animal experiments with animals in the supine position. Animals received no prior treatment. The feasibility and operation of closing mucosal defects with FLEXLOOP were investigated using ulcers 30 mm in diameter induced in the greater curvature of the middle stomach in the experimental animals. In addition, the operation time for closing mucosal defects using the ligating device with the snare by double‐channel endoscope as previously reported^15^ or FLEXLOOP with the polyamide/polyurethane tube was measured five times each by the same operating endoscopist. The operation time was set as the time from the insertion of the endoscope to its withdrawal. The number of clips used for closing the defect was set to five for each operation.

### Statistical analysis

All statistical analyses were performed using statistical software (JMP 14; SAS Institute Inc., Cary, NC, USA). Statistical differences were calculated by using the Wilcoxon signed‐rank test. A *p*‐value < 0.05 was considered statistically significant.

## RESULTS

### Bench testing

In the 1st prototype, the Roeder knot could not be slid at all. Therefore, in the 2nd prototype, the stopper was made of silicon rubber, and the material of the thread was changed to nylon. We could slide the stopper, but it was loose. Thus, the shape of the stopper was changed to a tighter conformation in the 3rd prototype. However, the stopper was still loose when it was slid, and the load on the stopper at both the start of sliding and at the start of loosening was low; thus, in the 4th prototype, the stopper was made of silicon rubber with a stainless steel pipe for tight adherence. This modification increased the load applied at the start of sliding and at the start of loosening, but the thread stretched greatly during sliding, and the force transmitted to the stopper when the loop was tightened was weak. For this reason, the 5th prototype was improved with a type of monofilament nylon thread connected to the middle of a stainless steel wire. As a result, we were able to develop the 5th prototype, which had a slightly lower load at the start of sliding than the conventional snare and an applied load when loosening the loop almost equal to that of the snare.

**FIGURE 4:**
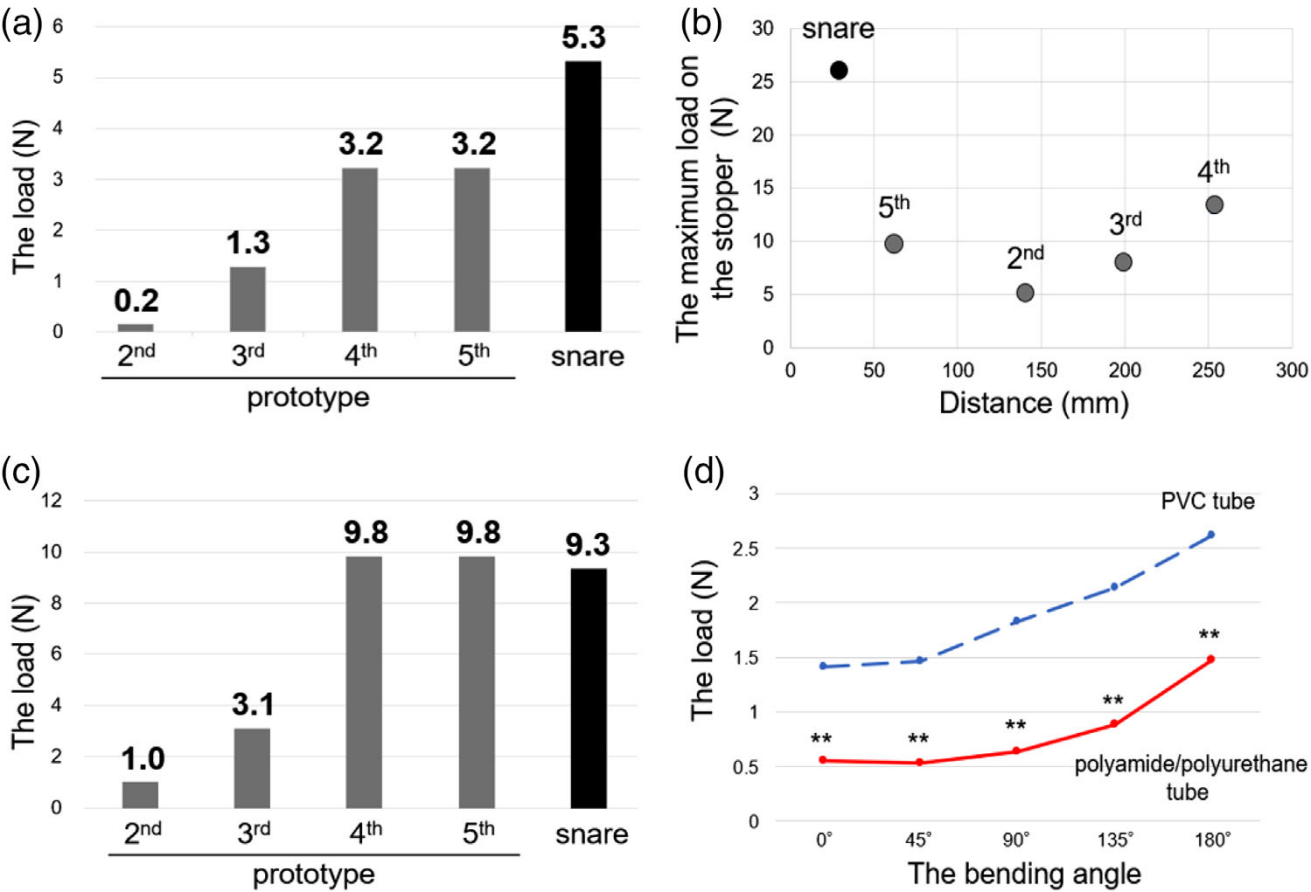
The magnitudes of the load on the stopper when starting to slide (a) and loosen the loop (b) are shown. Lower load on the stopper when starting means the device is easy to squeeze, and higher load on the stopper when loosening means the device is tight and kept closure force. (c) The magnitudes of the maximum load on the stopper and the distance of thread movement are shown. (d) The load on the outer sheath at all angles was significantly lower when the sheath was moved in the polyamide/polyurethane tube than when it was moved in the polyvinyl chloride (PVC) tube. **p < 0.01, All these experiments were measured five times, and the median values were described. Statistical differences were calculated using the Wilcoxon signed‐rank test

For each prototype, the magnitude of the load on the stopper when starting to tighten the loop is shown in Figure 4a. The magnitudes of the load on the stopper when starting to loosen the loop are indicated in Figure 4b. Additionally, the magnitude of the maximum load on the stopper and the distance of thread movement when the diameter of the sponge was decreased from 30 to 20 mm by squeezing are shown in Figure 4c. Finally, the magnitudes of the load on the outer sheath when starting to slide the sheath in the PVC tube or the polyamide/polyurethane tube were measured under the condition of bending the endoscope tip from 0 to 180°. The load on the outer sheath at all angles was significantly lower when the sheath was moved in the polyamide/polyurethane tube than when it was moved in the PVC tube (Figure 4d).

### Animal studies

**FIGURE 5:**
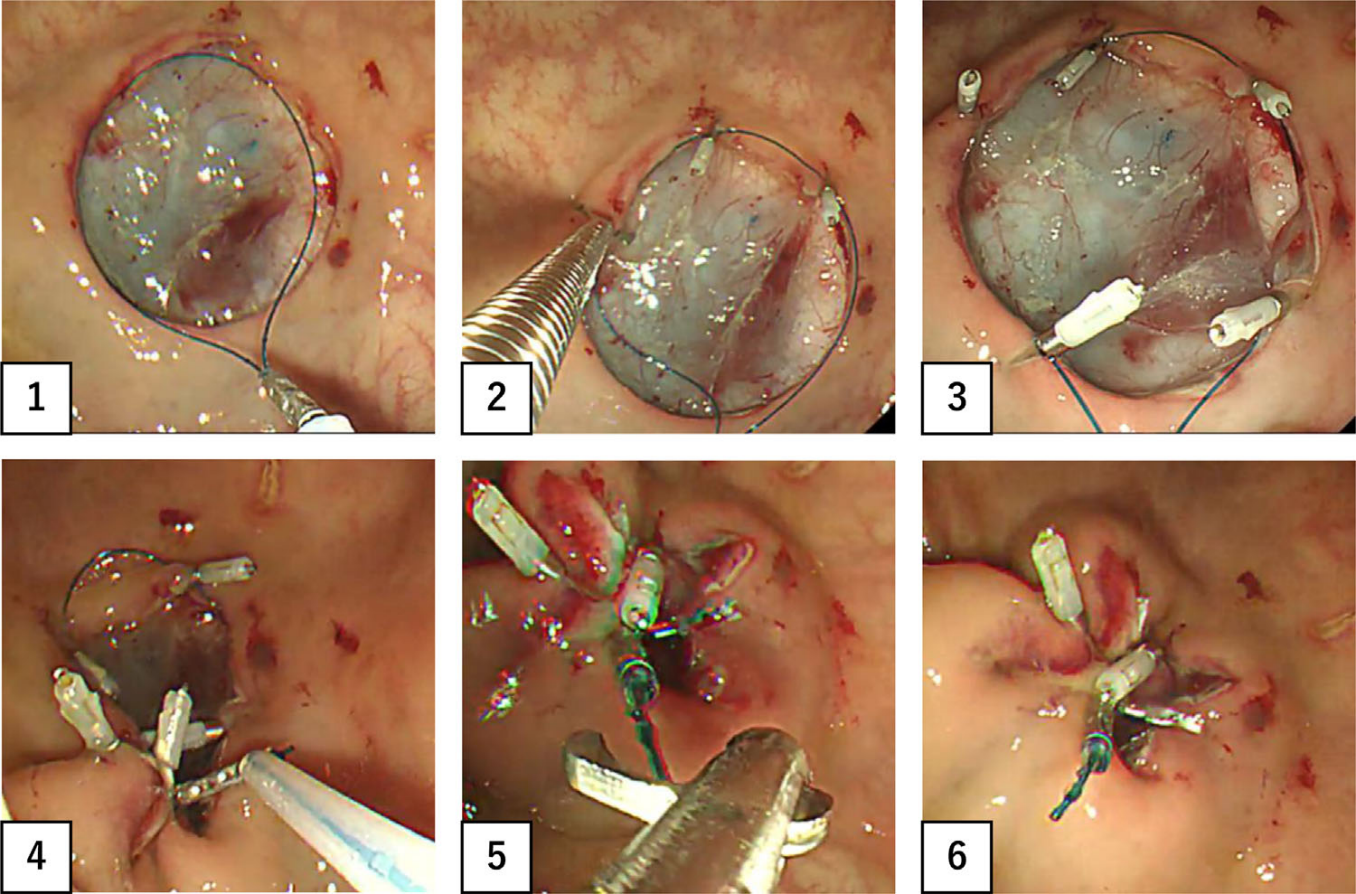
How to use FLEXLOOP in a pig is shown

**FIGURE 6:**
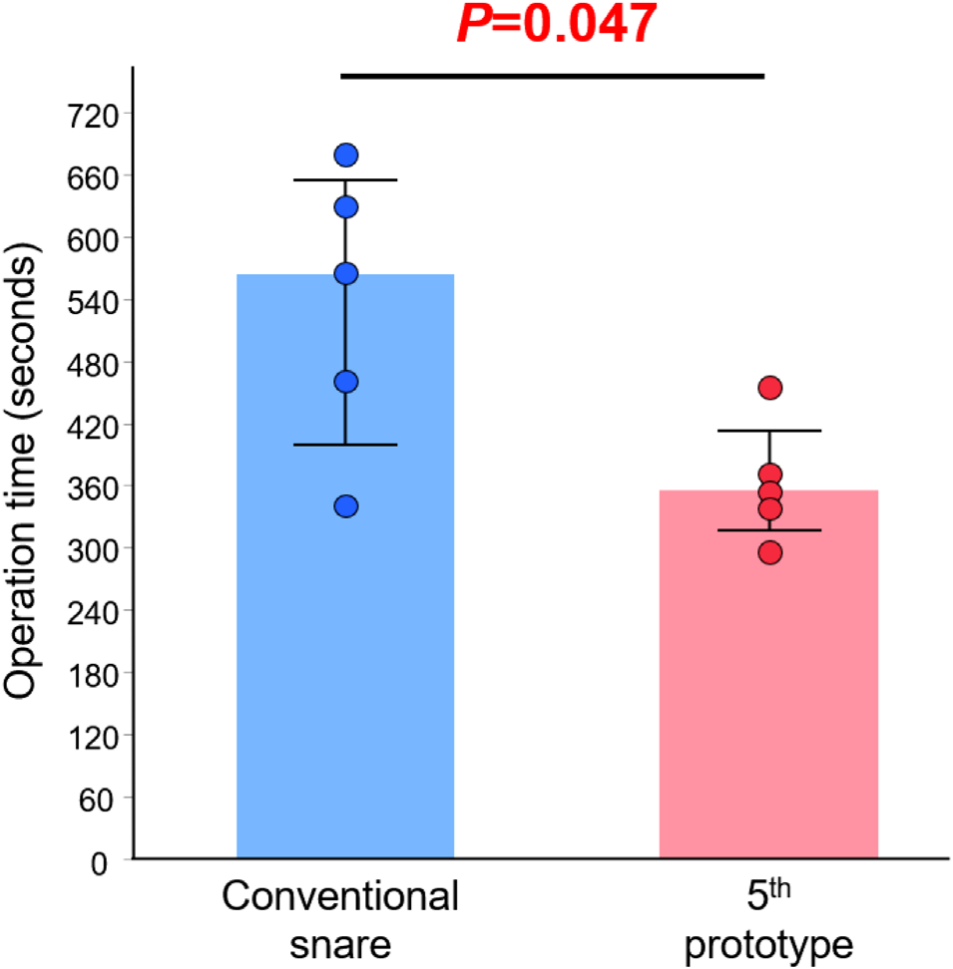
The median operation time for the five procedures was 563 s (range 340–679 s) with the conventional snare and 355 s (range 303–455 s) with FLEXLOOP^;^ the operation time was significantly shorter with FLEXLOOP than with the conventional snare (*p *= 0.047). Statistical differences were calculated using the Wilcoxon signed‐rank test

We confirmed the feasibility of mucosal defect closure with FLEXLOOP (Figure 5 and Video S1). With the conventional snare, when the endoscope tip was moved, the loop itself moved in the same direction. However, unlike the conventional snare, FLEXLOOP was less affected by the movement of the endoscope tip during the procedure, and the mucosal defect was closed smoothly after the anchor clip technique. Furthermore, the loop diameter could be reduced freely according to the size of the ulcer as necessary during the operation. Mucosal defects closure was successful all five times with both the snare and FLEXLOOP. The median operation time for the five procedures was 563 s (range 340–679 s) using the conventional snare and 355 s (range 303–455 s) with FLEXLOOP externally attached to the endoscope; the operation time was significantly shorter with FLEXLOOP than with the conventional snare (*p *= 0.047, Figure 6).

## DISCUSSION

The need for endoscopic mucosal defect closure has increased with the widespread use of ESD, the expansion of indications for elderly patients taking antithrombotic drugs, and the expansion of treatment indications to include more challenging sites, for example, the duodenum.

The conventional snare is not a dedicated device for closing mucosal defects and has not been optimized in terms of loop diameter, snare diameter, and stiffness. There are two ways to use the conventional snare: with a single‐channel endoscope^18^, ^19^ or with a double‐channel endoscope.^12^, ^13^, ^14^, ^15^, ^16^, ^17^ The features of these methods using the snare and FLEXLOOP are summarized in Table 1. Specifically, the single‐channel method requires reattachment of a snare tail and a hook device, which takes considerable time. On the other hand, the double‐channel method requires high technical skills, because the endoclips, loop, and optic axis move in the same direction. These methods have not been generalized due to these technical barriers.

**TABLE 1:** Feature of the conventional snare and FLEXLOOP

	Conventional snare		
	Single‐channel method	Double‐channel method	FLEXLOOP
Form	Two pieces (snare + ligating device)	Two pieces (snare + ligating device)	One piece
Endoscope	Single‐channel	Double‐channel	Single‐channel
Delivery	Through the scope	Through the scope	Externally attached to the scope
Usage	Use two pieces separately	Use two pieces without separating	Use as a single piece
Operability during anchoring with endoclips	◎	×	○
Unnecessity of reattachment	×	○	○
Unnecessity of cutting thread	○	○	×
Loop size adjustment during operation	×	×	○

◎ Excellent, ○ Good, × Inferior

The FLEXLOOP that we developed is a world‐first dedicated device for endoscopic mucosal defect closure, and its diameter, thickness, and tightening structure were optimized. The specifications were optimized by repeated bench testing and animal studies, and it was shown that both the performance and usability of the device were sufficient. Furthermore, we were able to confirm that the operation time was significantly shorter with FLEXLOOP than with the conventional snare. This can be explained by the observations that the loop is less subject to the movement of the endoscope tip, making it easier to operate and that our method has the advantage of the ability to reduce the size of the loop to fit the ulcer during the operation. These features would have made the anchor clip technique much easier and decreased the operation time. Additionally, due to the characteristics of FLEXLOOP, where the loop is less subject to the movement of the endoscope tip, there is a possibility that this device can be easily manipulated in a difficult location, such as the lesser curvature of the stomach. However, this was not investigated in this study, and further investigation is needed. Furthermore, in this study, we only examined the use of FLEXLOOP in the stomach. If the ulcer is within 30 mm, we believe this device can be applied to other sites such as the colon and duodenum. However, we have not actually studied the indications of this device on other sites. The feasibility of this device in other gastrointestinal tracts needs to be further investigated.

**FIGURE 7:**
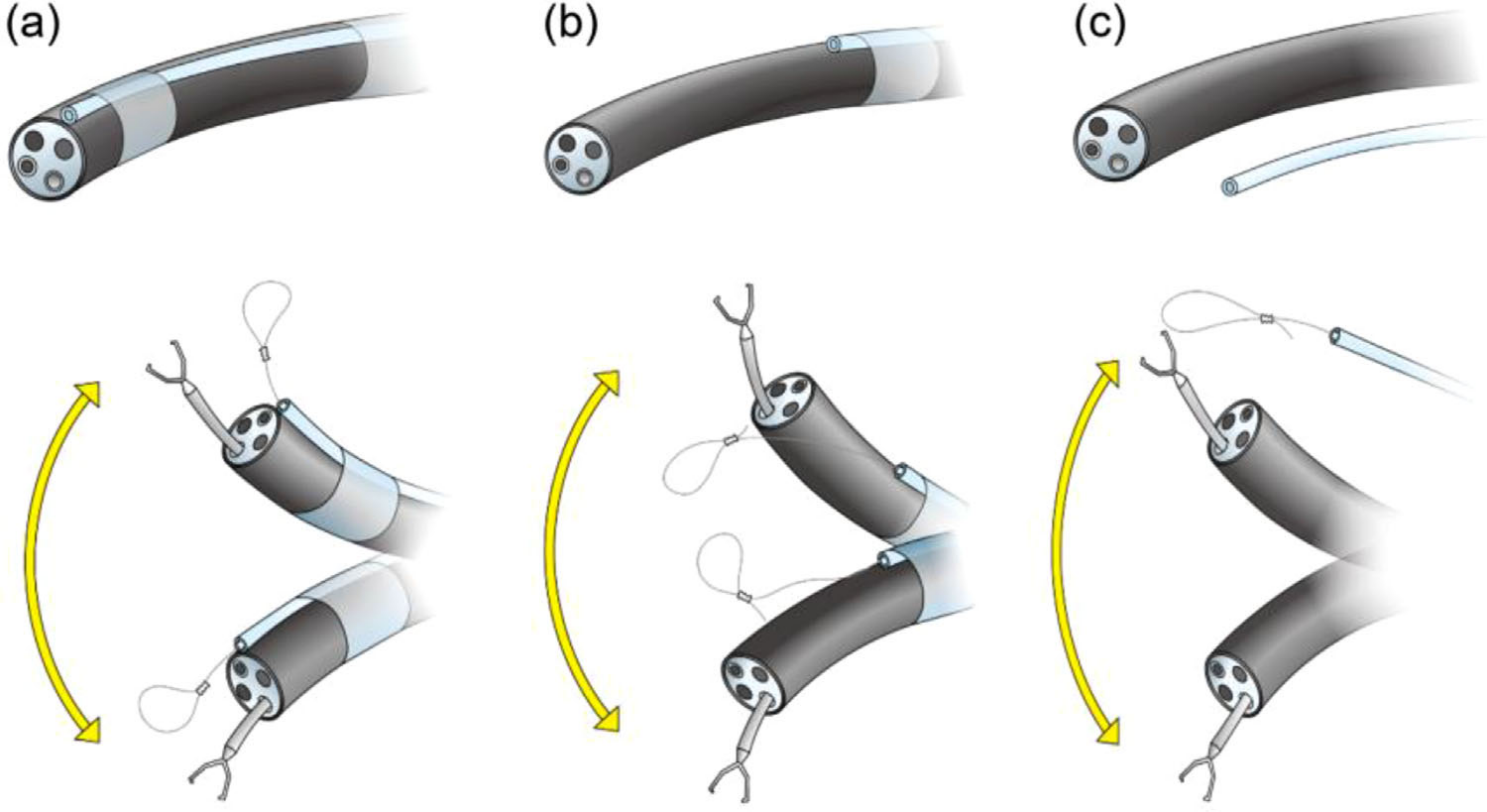
The outer tube was designed to be externally attached (a) to the tip of the endoscope or (b) in front of the flexure of the endoscope, or (c) to be completely independent of the endoscope

The outer tube was originally designed to be attached externally (a) to the tip of the endoscope or (b) in front of the flexure of the endoscope or (c) to be completely independent of the endoscope (Figure 7). When the endoscope and the device are used independently, as for the design described in (c), the synchronization of movement or “in‐line” motion between the endoscope tip and the loop can be reduced. On the other hand, there may be technical problems such as reduced operability of the loop and damage to organs. Specifically, the tip of the outer tube or sheath, which cannot be monitored, may damage the gastric mucosa unintentionally. In all the experiments in this study, FLEXLOOP was attached to the endoscope tip, and it is necessary to further examine the method of use in clinical practice in the future.

Recently, Inoue et al. reported a novel endoscopic purse‐string suture technique, “loop 9”, for gastrointestinal defect closure, and it took 14 min to complete the procedure.^21^ This method and ours are similar in the sense that the knot in the loop is pushed with the outer sheath and slides to tighten the loop, but there are some differences in the shape of the device and its usage. While the loop 9 technique has already been shown to be feasible and effective in a small number of actual patients, FLEXLOOP has not yet been used in a clinical setting and is currently being tested for preclinical use. As shown above, FLEXLOOP has been fully optimized as a dedicated device for the closure of mucosal defects, and it will be widely used in clinical practice for the purpose of defect closure.

We agree that this study has several limitations, as follows. First, this study was performed in a purely experimental animal setting, and because of the limited number of animals, we cannot exclude the possibility that our results are due to true biological variability. Second, we measured the load on the stopper when sliding the loop and the load applied when loosening the loop but were not able to examine other factors. Third, we cannot completely eliminate the operator bias because blind testing was impossible. In addition, it is necessary to study how many cases should be experienced to obtain the learning curve effect in actual use in the future. Fourth, the durability of FLEXLOOP in humans after suturing ulcers has not been studied. Fifth, we cannot compare the cost‐effectiveness because the price of the FLEXLOOP has not been decided yet.

In conclusion, FLEXLOOP can be a promising option for mucosal defect closure after gastric ESD.

## CONFLICT OF INTEREST

The entire experimental protocol of this study was approved by the institutional animal care and ethical review board (IVTeC Co. Ltd. Animal Welfare Committee). This study was performed in accordance with the Guide for the Care and Use of Laboratory Animals.

## ETHICS STATEMENT

The entire experimental protocol of this study was approved by the institutional animal care and ethical review board (IVTeC Co. Ltd. Animal Welfare Committee). This study was performed in accordance with the Guide for the Care and Use of Laboratory Animals.

## FUNDING INFORMATION

None.

## Supporting information


**Video S1**: A new method of endoscopic mucosal defect closure using FLEXLOOP.
